# Orientation analysis of pentacene molecules in organic field-effect transistor devices using polarization-dependent Raman spectroscopy

**DOI:** 10.1038/s41598-019-51647-2

**Published:** 2019-10-22

**Authors:** Bishwajeet Singh Bhardwaj, Takeshi Sugiyama, Naoko Namba, Takayuki Umakoshi, Takafumi Uemura, Tsuyoshi Sekitani, Prabhat Verma

**Affiliations:** 1 Department of Applied Physics, Osaka University, Suita, Osaka 565-0871 Japan; 2 The Institute of Scientific and Industrial Research, Osaka University, Mihogaoka, Ibaraki, Osaka 567-0047 Japan

**Keywords:** Raman spectroscopy, Electronic devices

## Abstract

Pentacene, an organic molecule, is a promising material for high-performance field effect transistors due to its high charge carrier mobility in comparison to usual semiconductors. However, the charge carrier mobility is strongly dependent on the molecular orientation of pentacene in the active layer of the device, which is hard to investigate using standard techniques in a real device. Raman scattering, on the other hand, is a high-resolution technique that is sensitive to the molecular orientation. In this work, we investigated the orientation distribution of pentacene molecules in actual transistor devices by polarization-dependent Raman spectroscopy and correlated these results with the performance of the device. This study can be utilized to understand the distribution of molecular orientation of pentacene in various electronic devices and thus would help in further improving their performances.

## Introduction

Organic field-effect transistors are recognized as important devices for their applications in several electronic systems such as digital screens^1^, electronic papers^2^, plastic circuits^3^, price tags^4^, non-volatile plastic memories^5^ and sensors^6^ due to their lightweight, low cost production, mechanical flexibility as well as better carrier mobility. Among various organic materials, pentacene has been extensively investigated as a promising organic material for transistor devices^7,8^ and finds application in various devices such as in memory device^9^, ambipolar transistor device^10^, and synaptic device^11,12^. The carrier mobility of pentacene depends upon various factors, such as, the molecular orientation, the grain size within the device and the presence of impurities. The molecular orientation has a profound impact on the charge transport and determines charge carrier mobility of the electronic devices. Any difference of orientation between adjacent pentacene molecules would degrade the interactions of π electrons between them, which would subsequently affect the flow of charge carriers within the device. Although extensive research exists on the influence of impurities and morphology of pentacene film on device performance^13–18^, investigation of the effect of molecular orientation on the performance of a transistor device has not been reported. Nevertheless, it is of great importance to understand the correlation between the orientation of pentacene molecules and the electronic properties of a transistor device for improving the performance of pentacene-based transistor devices.

Raman spectroscopy has been recognized as a powerful technique to investigate structural features of organic molecules with a reasonably high spatial resolution of about 200~300 nm^19,20^. The resolution can be further improved to a few nanometers by utilizing plasmonic properties of metallic nanotip in tip-enhanced Raman spectroscopy technique^21–26^. Raman spectroscopy is also advantageous over other techniques because certain molecular vibrational modes can be preferentially invoked by employing a particular polarization of the incident light, and hence Raman spectroscopy can be utilized to investigate orientation distributions of various molecules, including pentacene^27–30^. In conventional Raman spectroscopy, the polarization measurements are limited to the direction parallel to the substrate plane, which usually suppress the molecular vibrations in the direction perpendicular to the sample plane. However, in our previous research, we investigated the orientation of pentacene molecules standing close to the normal on a substrate by utilizing a unique control of the polarization, where a component of the incident light could be polarized in a direction perpendicular to the substrate plane^31^. We utilized incident light with two different polarizations, namely radial and azimuthal polarizations, the former being perpendicular to the substrate and the later in a direction parallel to the substrate. The pentacene molecule has an in-plane vibrational mode, namely the B_3g_ mode, at 1596 cm^−1^ that has atomic displacements along its long molecular axis. If pentacene molecules are standing on a substrate, atomic displacements of the B_3g_ mode would be in the direction perpendicular to the substrate. Therefore, Raman intensity of this mode would be maximum for the radially polarized incident light, while it would be minimum for the azimuthally polarized incident light. On the other hand, if the pentacene molecules lie on the substrate, the radial polarization of incident light would result in minimum Raman intensity, while azimuthal polarization of incident light would show maximum Raman intensity of B_3g_ mode. Therefore, if the pentacene molecules are oriented at a certain angle from the normal, the ratio of Raman intensities for B_3g_ mode under these two polarizations would give information about the tilt angle of the pentacene molecules. Compared with Raman spectroscopy, other existing characterization techniques have several limitations. For example, atomic force microscopy (AFM) can be utilized to investigate the morphology of the molecules but not their orientations. The information about molecular orientation of pentacene can also be obtained from X-ray diffraction spectroscopy (XRD) technique, however, this technique has very low spatial resolution, which only gives an average value of orientation over a large area and hence it is not suitable for investigating the effect of molecular orientations on carrier mobility. Also, this technique cannot be applied to study disordered part of the sample. Therefore, Raman spectroscopy is the only tool that can provide us information on molecular orientation nondestructively at a reasonably high spatial resolution within a transistor device.

In this study, we have investigated the orientation of pentacene molecules in actual transistor devices by utilizing polarization-dependent Raman spectroscopy and correlated these results with the performance of the device. In order to understand the effect of molecular orientation on electronic performance of the device, we used two different types of pentacene-based transistors, namely the sublimated and the non-sublimated. The as-grown pentacene molecules are considered as non-sublimated because they contain some impurities, such as 6,13-dihydropentacene, 6,13- pentacenequinone, aluminum, and iron. Such non-sublimated molecules can be purified by a process known as sublimation to remove all impurities and obtain a sublimated sample with almost no impurity. We measured electrical characteristics of the devices fabricated from both kind of pentacene molecules and found that the sublimated transistor showed higher charge carrier mobility than the non-sublimated. The impurity molecules can affect the device performance in two ways. One, they disturb the uniformity of pentacene molecules on the active layer of the device because of their presence and thus degrade the carrier mobility in the device. Second, since the impurity molecules go between the pentacene molecules, they push the pentacene molecules from their original orientations, as a result of which the orientations of pentacene molecules can randomly change on the substrate. Since even a very little change in the orientation of neighboring molecule can significantly affect the overlap of π electrons of the molecule, it drastically affects the carrier mobility in the device. The later effect can be much more prominent than the former, and hence it is important to understand the variation of molecular orientation between neighboring pentacene molecules in a non-sublimated device and to compare the same with a sublimated device. In order to correlate the device performance with the presence of impurity molecules in a non-sublimated device as compared to a sublimated device, we quantitatively investigated the distributions of molecular orientation of pentacene molecules in both the devices using polarization-dependent Raman spectroscopy. Our results show that the variation of orientation between adjacent measurement points, separated by a distance of a few hundred nanometers, was smaller in the device with sublimated pentacene molecules as compared to the non-sublimated device. This suggests that the flow of carriers in the sublimated sample was better in comparison with the non-sublimated sample due to a comparatively better molecular orientation uniformity in the sublimated sample. Also, our analysis confirms that the pentacene molecules in the sublimated sample were aligned in wider areas in comparison with those in the non-sublimated device, and therefore the sublimated device shows higher charge carrier mobility.

## Results and Discussion

**Figure 1:**
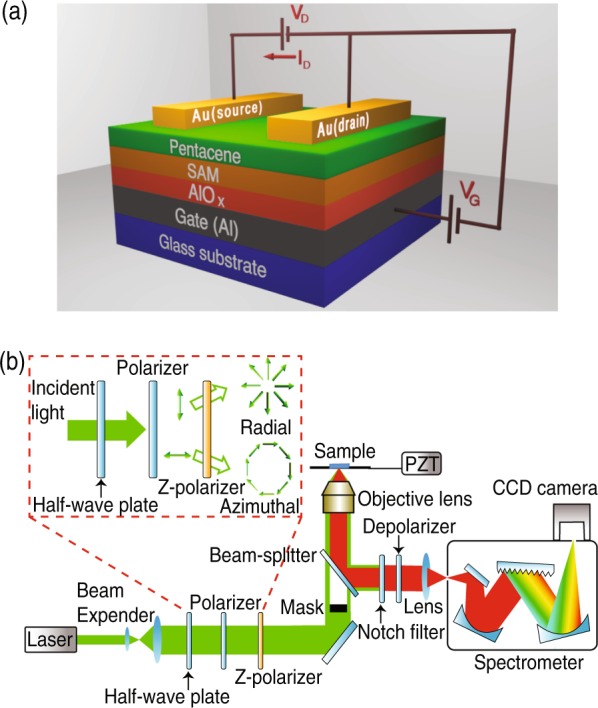
(**a**) Schematic of the fabricated pentacene transistor device, and (**b**) optical setup for Raman spectroscopy measurement.

**Figure 2:**
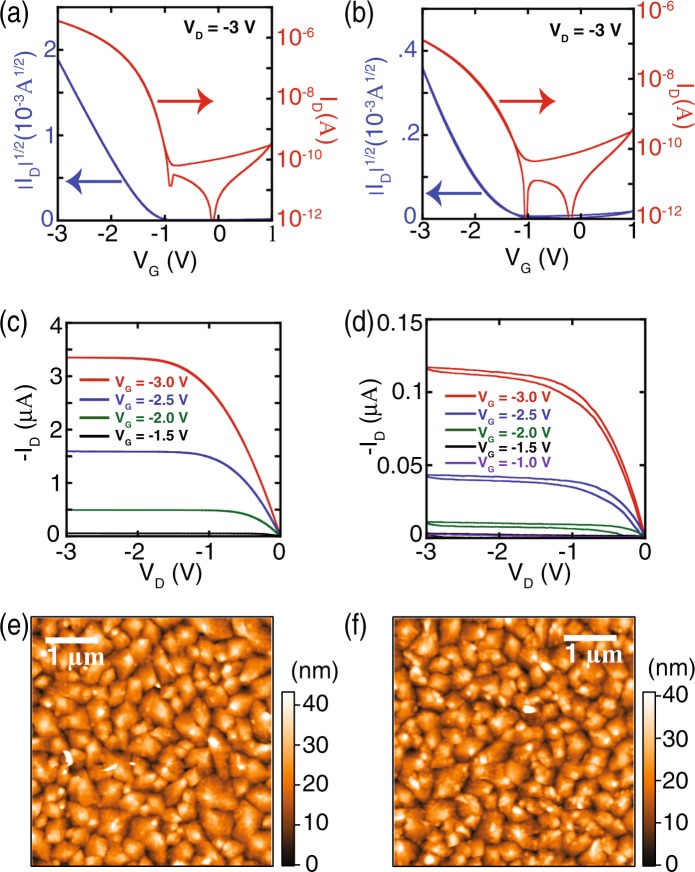
Transfer curves of the (**a**) sublimated and (**b**) non-sublimated pentacene transistor devices. Output characteristics of the (**c**) sublimated and (**d**) non-sublimated pentacene transistor devices. AFM images of the surface morphologies of the (**e**) sublimated and (**f**) non-sublimated pentacene devices.

In order to confirm the electronic performance, we connected the fabricated device with gate-source voltage (V_G_) and drain-source voltage (V_D_), as shown in Fig. 1(a), and measured electrical properties of the fabricated OFET devices. The transfer curve and output characteristics of the devices for the sublimated and the non-sublimated pentacene transistors are shown in Fig. 2. The transfer curves of the devices were monitored by changing V_G_ from 1.0 to −3 V by keeping the value of V_D_ at −3 V. The results are shown in Fig. 2(a,b) for sublimated and non-sublimated devices, respectively. We changed V_G_ from −1.0 to −3 V in steps of 0.5 V and monitored drain-source current (I_D_) of the transistor device as a function of V_D_, as shown in Fig. 2(c,d) for the sublimated and the non-sublimated devices, respectively. The calculated values of carrier mobilities for the sublimated and the non-sublimated pentacene transistors device were measured to be 0.603 cm^2^/Vs and 0.0352 cm^2^/Vs, respectively. Thus, the value of the carrier mobility for the sublimated device was found to be 17.1 times higher than that of the non-sublimated device.

The grain morphologies of pentacene may have significant effect on the charge carrier mobilities^13^. Thus, we investigated grain morphologies using AFM technique. Figure 2(e,f) show typical AFM images that represent grain morphologies of the sublimated and the non-sublimated pentacene devices, respectively. We observed that the surface morphologies in both devices appeared similar with negligible changes in grain sizes. Therefore, we conclude that the grain size or the surface morphology in our devices are not noticeably affected by the sublimation and hence have negligible effect on the electronic performance of the devices. In general, AFM is a powerful and versatile technique that gives various information about the quality and surface morphology of the pentacene thin film, which affect the performance of the transistor device. However, since the AFM images of the sublimated and non-sublimated devices in present devices did not show an obvious difference, we need to rely on Raman spectroscopic analysis of both the devices to get further insight into the reasons for the high carrier mobility for the sublimated device.

**Figure 3:**
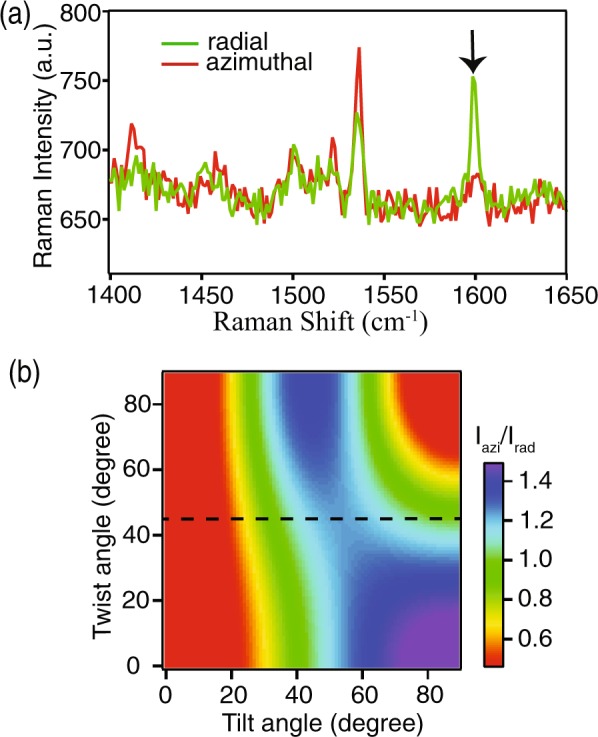
(**a**) Typical Raman spectra of pentacene molecules measured with incident power of 120 µW and exposure time of 3 s. The red and green color spectra show Raman spectrum measured with azimuthal and radial polarizations, respectively, at the same sample position. The arrow indicates the B_3g_ mode utilized for investigating the molecular orientations. (**b**) Theoretically calculated dependence of the intensity ratio on tilt and twist angles.

Figure 3(a) shows typical Raman spectra of pentacene molecules with radial and azimuthal polarization of incident light. We observed that the Raman intensity at 1596 cm^−1^, which corresponds to the B_3g_ vibrational mode of pentacene^31^, drastically increased for the radially polarized incident light as compared to the azimuthally polarized incident light. Although a polarization dependence can be seen for other vibrational modes, such as for the mode at 1371 cm^−1^ and 1533 cm^−1^, the B_3g_ mode at 1596 cm^−1^ is found to be more sensitive to the incident polarization. Therefore, we selected this mode for further analysis. The choice of the B_3g_ mode would ensure minimum error due to higher contrast between the two polarizations in comparison to the other modes. The ratio of Raman intensities for the polarization-sensitive B_3g_ vibrational mode, measured with azimuthal and radial polarizations at the same position, enables us to evaluate the molecular orientation by estimating the tilt angle of pentacene molecules in the device^31^.

In order to obtain information about the tilt angle, we converted the intensity ratios for the two incident polarizations to the orientation angles by considering various parameters for calculation. The collection efficiencies of the objective lens for the parallel and the perpendicular components of the incident polarization, A and B, respectively, in the backscattering configuration depend on its cone angle^30^. In our experiment, we used an objective lens (NA = 0.95) that has a cone angle of 71.8° and obtained the values of A and B as 9.98 and 1.11, respectively. In general, the pentacene molecules can have a tilt with respect to its vertical position and at the same time, molecules can be twisted along the molecular axis. We can estimate the tilt and the twist angles by considering the collection efficiencies of objective lens and measured Raman intensities of the B_3g_ vibrational mode under azimuthal and radial polarizations^31^. The calculated dependence of Raman intensity ratios on both, the tilt angle and the twist angle, are shown in Fig. 3(b). From the color scale in Fig. 3(b), one can realize that there are several possible combinations of the tilt angle and the twist angle for a given value of the intensity ratio. Since it is known that the pentacene molecules tend to stand nearly perpendicular on the AlO_X_/SAM substrate^32^, we can ignore the larger value of tilt angles that are shown on the right-half of the color image in Fig. 3(b). Thus, by considering only the left-half part of the figure, one can realize that contribution of twist angle is not significant and the intensity ratio dominantly depends on the tilt angle of pentacene. Hence, in order to understand the influence of molecular tilt, we fixed the twist angle at 45° to determine the tilt angle, which is indicated by the dotted line in Fig. 3(b). By considering this method, we utilized Fig. 3(b) as the reference to calculate the tilt angle for any measured Raman intensity ratio and then constructed images of the distribution of molecular tilt orientation for both transistor devices. This measurement was performed in a 10 × 10 µm^2^ area of the device with 30 × 30 pixels. Our measurement at each position provides the average tilt angle of pentacene molecules within the focal volume of the incident laser, which is a few hundred nanometers. Here, it is noteworthy to mention that the molecules within the focal volume may still have different orientations, and it is not possible to estimate the orientation of individual molecules. Nevertheless, Raman spectroscopy provides us the information about average tilt angle of pentacene molecules with a high spatial resolution of a few hundred nanometers in a transistor device, which is not possible with other techniques.

**Figure 4:**
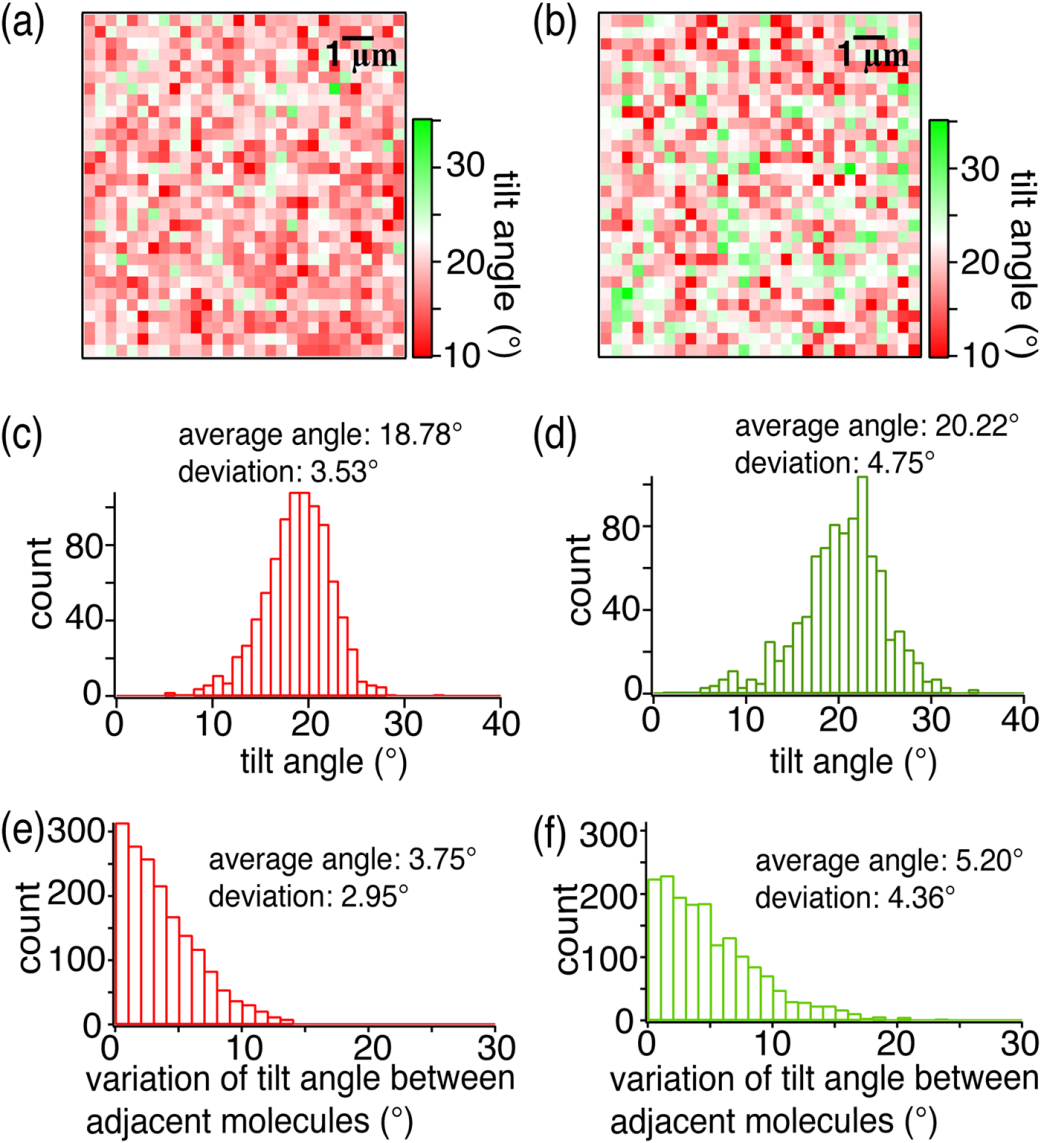
Molecular tilt orientation distribution calculated from Raman intensity ratio of azimuthal to radial polarization of incident light for (**a**) sublimated and (**b**) non-sublimated pentacene transistors. Histograms showing tilt angle variation for (**c**) sublimated and (**d**) non-sublimated pentacene transistors, and histograms showing the variation of tilt angle between adjacent pixels for (**e**) sublimated and (**f**) non-sublimated pentacene transistors.

Figure 4(a–d) show the distribution of molecular tilt orientation of pentacene molecules for both the transistor devices along with the histograms of the tilt angles. From the histogram, the average value of tilt angles for the sublimated and the non-sublimated devices were estimated to be 18.78° and 20.22°, respectively. The histogram of the tilt angles for the sublimated device, shown in Fig. 4(c), depicts narrower span ranging over about 20° in comparison to that for the non-sublimated device shown in Fig. 4(d), which spans over a range of about 30°. This decrease in the variation in tilt angle for sublimated device is also evident from its lower standard deviation of ±3.53° in comparison to that for the non-sublimated device that was estimated to be ±4.75°. We found that the average value of the tilt angle and the deviation for the sublimated device were smaller than those for the non-sublimated pentacene device, which enables efficient transport of charge carrier from source to drain in the sublimated pentacene transistor device. In fact, the smaller deviation in tilt angle for the sublimated device in comparison to the non-sublimated device is the dominant reason for better carrier mobility as small difference in average tilt angle between the two devices is not expected to drastically affect the carrier mobility. The presence of impurities in the non-sublimated pentacene can be a reason for its larger deviation in tilt angles. During the vacuum deposition of non-sublimated pentacene, molecules of the impurities can get trapped between or beneath the pentacene molecules which, can randomly change their tilt angles and significantly affect the mobility of charge carriers.

As observed before by electronic measurements, the value of the carrier mobility for the sublimated device was much higher than that for the non-sublimated device. However, the difference between average tilt angle for the sublimated and the non-sublimated pentacene device was only 1.44 degree. Therefore, to get a better insight into the large difference of carrier mobility of the two devices, we further analyzed the variation of tilt angle difference among all the adjacent measurement positions in the device by taking advantage of the high spatial resolution of our Raman measurements. For this purpose, we subtracted the tilt angle at any particular position from the tilt angle at its adjacent position, which is separated by a few hundred nanometers in the device, and created a histogram map that shows the difference of tilt angles among the adjacent pixels in the devices as shown in Fig. 4(e,f) for the sublimated and non-sublimated devices, respectively. It is clear from these histograms that there are more number of positions where angle differences between adjacent measurement points are smaller in the case of sublimated pentacene device as compared to that of non-sublimated device. We obtained the value of the average angle difference for the sublimated and the non-sublimated devices as 3.75° ± 2.95° and 5.20° ± 4.36°, respectively. Thus, we found that the average angle variation between adjacent points was smaller for the sublimated device with smaller standard deviation. When adjacent molecules have larger tilt angle difference between them, the interactions of their π electrons is degraded, and hence the smaller difference in angles between the adjacent positions enables efficient transfer of charge carriers from one position to its neighboring position. This results in a relatively higher carrier mobility for the sublimated device as compared to the non-sublimated device. In order to confirm the uniformity of our results, we tested four additional OFETs devices, two sublimated and two non-sublimated. We found that the sublimated devices showed comparatively smaller average tilt angles with smaller deviations and smaller differences in tilt angles between the adjacent positions in comparison to the non-sublimated devices. We can thus confirm that our observation about the molecular tilt and its variation between sublimated and non-sublimated devices was uniform across several devices.

**Figure 5:**
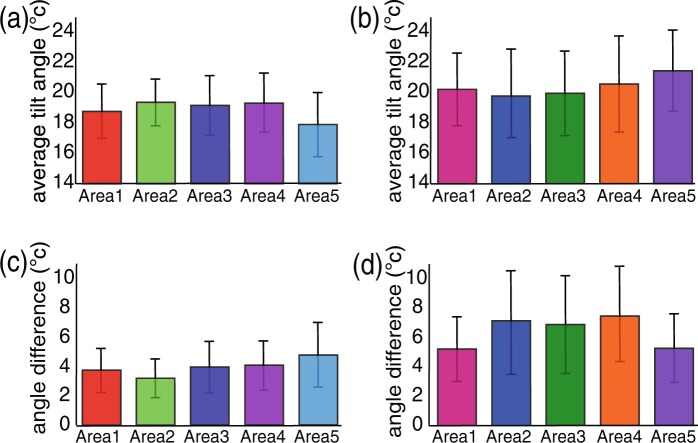
Bar graphs show average of orientation tilt angles at five different areas of the devices with the standard deviations for (**a**) sublimated and (**b**) non-sublimated pentacene transistor devices, Average angle difference between the adjacent pixels at the measured areas of the devices for (**c**) sublimated pentacene and (**d**) non-sublimated pentacene transistor devices.

In addition, similar measurements were performed in multiple areas of the devices to confirm whether or not the same properties are responsible for the efficient transfer of charge carriers in wider areas of the devices. Figure 5(a,b) show the average tilt angle in five different areas (10 µm × 10  μm each) of the sublimated and the non-sublimated device, respectively, along with their standard deviation bars. The measurement areas were separated from each other by a distance of 10 µm in the active region of the device and their corresponding orientation images are shown in the Supplementary Information. The values of average tilt angles were 18.78° ± 3.53°, 19.36° ± 3.05°, 19.16° ± 3.89°, 19.34° ± 3.87°, and 17.90° ± 4.18° at five different areas of the sublimated device. On the other hand, we observed the average tilt angles to be 20.22° ± 4.75°, 19.40° ± 5.77°, 19.96° ± 5.53°, 20.56° ± 6.20°, and 21.46° ± 5.32° at five different areas of the non-sublimated pentacene device that show comparatively larger orientation angles with larger standard deviations than those of the sublimated device. Figure 5(c,d) represent the average angle differences between the adjacent pixels in these five measured areas of both the devices. Here, the sublimated device shows smaller values of average of angle differences with smaller standard deviations in all the measured areas compared to those for the measurement areas of the non-sublimated device. Thus, we conclude that the sublimated pentacene molecules are better aligned in wider areas of the device compared to the non-sublimated pentacene molecules, which contributes to its high charge carrier mobility.

## Conclusions

This work has demonstrated an analytical investigation of orientation angles of pentacene in transistor devices. The transistor devices fabricated using sublimated and non-sublimated pentacene molecules show large differences in their carriers mobilities. Although the morphologies of pentacene in both the devices appear similar by AFM analysis, we found that the average orientation angle and the angle differences among adjacent positions separated by few hundred nanometers were smaller for the sublimated transistor device in comparison to the non-sublimated device, which was estimated from a polarization-dependent Raman spectroscopy technique. We further investigated orientation distributions of pentacene in wider areas of both the devices. The results show comparatively smaller orientation angle with small deviation for the sublimated pentacene device in different measured areas than the non-sublimated device. The difference of orientation angles among adjacent positions were also smaller for the sublimated pentacene device in all measured areas. Therefore, the carrier mobility for the sublimated device is significantly better than that for the non-sublimated device. This work demonstrates a way to quantitatively investigate orientation of pentacene molecules in electronic devices using a non-destructive and non-invasive Raman technique capable of offering a reasonably high spatial resolution of a few hundred nanometers, which can be utilized to improve performance of the device.

## Experimental Methods

### Materials

Both sublimated (P2524, 99.999%) and non-sublimated pentacene molecules were received from Tokyo Chemical Industry Co., Ltd., Japan. They were then utilized to fabricate the sublimated and non-sublimated devices.

### Fabrication of transistor devices

Figure 1(a) shows a schematic of a fabricated transistor device. We first deposited a 30-nm-thick aluminum (Al) layer on a glass substrate (Corning Eagle XG, Germany) using vacuum deposition (EX-200, ULVAC, Japan). The substrate was then anodized in 1 mM of citric acid to grow an aluminum oxide (AlO_x_) layer. In the next step, the substrate was immersed in 3 mM of dodecylphosphonic acid (C_12_H_27_O_3_P) for 12 h at 30 °C, followed by rinsing in isopropyl alcohol and drying with nitrogen gas that produced a self-assembled monolayer (SAM) of dodecylphosphonate on the substrate. The fabrication process was followed by a vacuum deposition of a 30-nm-thick layer of pentacene molecules, which was deposited at the rate of 0.2 Å/s. Finally, a 50-nm-thick gold layer was deposited on these substrates through a shadow mask to fabricate source/drain contacts followed by an annealing of the device at 100 °C for 1 h under vacuum. The channel length and width of the transistor device were 40 µm and 500 µm, respectively. We fabricated two devices using sublimated and non-sublimated pentacene molecules under the same fabrication conditions.

### Electronic measurements

The current-voltage characteristics of pentacene transistors were measured with a semiconductor parameter analyzer (B1500A, Keysight Technologies, United States) and a probe station.

### Atomic force microscope (AFM) measurements

The AFM images of the device were measured in the tapping-mode operation using AFM5000II (Hitachi, Japan).

### Raman measurement

Figure 1(b) illustrates an optical setup for Raman spectroscopy measurements in backscattering configuration. An excitation laser with a wavelength of 532 nm, which meets off resonant condition for pentacene molecule, was tightly focused on the sample plane through a 150× objective lens (NA = 0.95). The laser light passed through a half-wave plate, a linear polarizer, a z-polarizer and a circular mask before it was focused on the sample. The combination of a half-wave plate and a polarizer controls the direction of linear polarization of the incident light. By changing direction of linear polarization of incident light, from horizontal to vertical, and by introducing a z-polarizer in the optical path, we generated azimuthal and radial polarizations at the sample plane, as illustrated in the inset of Fig. 1(b). A strongly dominant perpendicular polarization of excitation at the sample plane was achieved by using a circular mask in the optical path that blocks light with small NA (<0.8). Raman scattered light from the sample was collected by the same objective lens, which was then passed through a depolarizer (DEQ-2S, Opto Sigma, Japan) and dispersed with a spectrograph (Princeton Instruments, Acton SP2300, Acton, USA) equipped with a grating (1800 grooves/mm), and was detected using an EM-CCD camera (Princeton Instruments, Pixis 100, Acton, USA). A notch filter was utilized to efficiently block Rayleigh scattered light.

## Supplementary information

supplementary information

## References

[1] Rogers JA (2001). Paper-like electronic displays: Large-area rubber- stamped plastic sheets of electronics and microencapsulated electrophoretic inks. Proc. Natl. Acad. Sci. USA.

[2] Eder F (2004). Organic electronics on paper. Appl. Phys. Lett..

[3] Crone BK (2001). Design and fabrication of organic complementary circuits. J. Appl. Phys..

[4] Pannemann C, Diekmann T, Hilleringmann U (2003). Organic Field-Effect-Transistors with Pentacene for radio-controlled-price-tag applications. Advances in Radio Science.

[5] Chi H-Y, Hsu H-W, Tung S-H, Liu C-L (2015). Nonvolatile Organic Field-Effect Transistors Memory Devices Using Supramolecular Block Copolymer/Functional Small Molecule Nanocomposite Electret. ACS Appl. Mater. Interfaces.

[6] Someya T (2005). Conformable, flexible, large-area networks of pressure and thermal sensors with organic transistor active matrixes. Proc. Natl. Acad. Sci. USA.

[7] Lin Y-Y, Gundlach DJ, Nelson SF, Jackson TN (1997). Pentacene-based organic thin-film transistors. IEEE Trans. Electron Devices..

[8] Kelley TW (2003). High-Performance OTFTs Using Surface-Modified Alumina Dielectrics. J. Phys. Chem. B.

[9] Lv Z (2019). Mimicking Neuroplasticity in a Hybrid Biopolymer Transistor by Dual Modes Modulation. Adv. Funct. Mater..

[10] Zhou Y, Han S-T, Xu Z-X, Roy VAL (2012). Controlled Ambipolar Charge Transport Through a Self- Assembled Gold Nanoparticle Monolayer. Adv. Mater..

[11] Wang Y (2018). Photonic Synapses Based on Inorganic Perovskite Quantum Dots for Neuromorphic Computing. Adv. Mater..

[12] Ren Y (2018). Gate-Tunable Synaptic Plasticity through Controlled Polarity of Charge Trapping in Fullerene Composites. Adv. Funct. Mater..

[13] Gomar-Nadal E, Conrad BR, William GC, Willams ED (2008). Effect of Impurities on Pentacene Thin Film Growth for Field-Effect Transistors. J. Phys. Chem. C.

[14] Jurchescu OD, Baas J, Palstra TTM (2004). Effect of impurities on the mobility of single crystal pentacene. Appl. Phys. Lett..

[15] Shtein M, Mapel J, Benzinger JB, Forrest SR (2002). Effects of film morphology and gate dielectric surface preparation on the electrical characteristics of organic-vapor-phase-deposited pentacene thin-film transistors. Appl. Phys. Lett..

[16] Knipp D, Street RA, Völkel A, Ho J (2003). Pentacene thin film transistors on inorganic dielectrics: Morphology, structural properties, and electronic transport. J. Appl. Phys..

[17] Knipp D, Street RA, Völkel AR (2003). Morphology and electronic transport of polycrystalline pentacene thin-film transistors. Appl. Phys. Lett..

[18] Ruiz R, Papadimitratos A, Mayer AC, Malliaras GG (2005). Thickness Dependence of Mobility in Pentacene Thin-Film Transistors. Adv. Mater..

[19] Bhardwaj BS (2019). Raman Spectroscopic Studies of Dinaphthothienothiophene (DNTT). Materials.

[20] Rodriguez-Martinez X (2017). Quantifying local thickness and composition in thin films of organic photovoltaic blends by Raman scattering. J. Mater. Chem. C.

[21] Verma P (2017). Tip-Enhanced Raman Spectroscopy: Technique and Recent Advances. Chem. Rev..

[22] Verma P (2006). Near-field Raman scattering investigation of tip effects on C60 molecules. Phys. Rev. B.

[23] Okuno Y, Saito Y, Kawata S, Verma P (2013). Tip-Enhanced Raman Investigation of Extremely Localized Semiconductor-to-Metal Transition of a Carbon Nanotube. Phys. Rev. Lett..

[24] Saito Y (2009). Nano-scale analysis of graphene layers by tip-enhanced near-field Raman spectroscopy. J. Raman Spectrosc..

[25] Matsui R (2007). Nanoanalysis of crystalline properties of GaN thin film using tip-enhanced Raman spectroscopy. Appl. Phys. Lett..

[26] Yano T (2013). Tip-enhanced nano-Raman analytical imaging of locally induced strain distribution in carbon nanotubes. Nat. Commun..

[27] Basova TV, Kolesov BA (1998). Raman polarization studies of the orientation of molecular thin films. Thin Solid Films.

[28] Presser V (2009). Raman polarization studies of highly oriented organic thin films. J. Raman Spectrosc..

[29] Stenger I (2009). Polarized micro-Raman spectroscopy study of pentacene thin films. Appl. Phys. Lett..

[30] Saito Y, Verma P (2012). Polarization-Controlled Raman Microscopy and Nanoscopy. J. Phys. Chem. Lett..

[31] Mino T (2012). Molecular orientation analysis of organic thin films by z-polarization Raman microscope. J. Raman Spectrosc..

[32] Fukuda K (2010). Thermal stability of organic thin-film transistors with self-assembled monolayer dielectrics. Appl. Phys. Lett..

