# Supplementary material for: Orientation analysis of pentacene molecules in organic field-effect transistor devices using polarization-dependent Raman spectroscopy
Source: Sci Rep. 2019 Oct 22;9:15149. doi: 10.1038/s41598-019-51647-2 (PMC6805883; doi:10.1038/s41598-019-51647-2)
Supplement: Supplementary file 1 — supplementary information [file 41598_2019_51647_MOESM1_ESM.docx]

**Supplementary information**

**Orientation analysis of pentacene molecules in organic field-effect transistor devices using polarization-dependent Raman spectroscopy**

Bishwajeet Singh Bhardwaj^1,a^, Takeshi Sugiyama^1,a^, Naoko Namba^2^, Takayuki Umakoshi^1^, Takafumi Uemura^2^, Tsuyoshi Sekitani^2^ and Prabhat Verma^1,^*

^1^Department of Applied Physics, Osaka University, Suita, Osaka 565-0871, Japan.

^2^The Institute of Scientific and Industrial Research, Osaka University, Mihogaoka, Ibaraki, Osaka 567-0047, Japan.

*****Correspondence: verma@ap.eng.osaka-u.ac.jp; Tel.: +81-6-6879-4710

^a^These authors contributed equally.

Figures S1 and S2 show the distribution of molecular tilt orientation of the pentacene molecules along with histograms of their tilt angles and histograms of variation of the tilt angles between adjacent measurement points with a separation of a few hundred nanometers in five different areas (10 µm $\times$ 10 µm each) of the sublimated and the non-sublimated devices, respectively. The measurement areas were separated from each other by a distance of 10 µm in the active region of both the devices. While values of average tilt angles were found to be 18.78°±3.53°, 19.36°±3.05°, 19.16°±3.89°, 19.34°±3.87°, and 17.90°±4.18° for five different areas of the sublimated device, those for the non-sublimated device were found to be 20.22°±4.75°, 19.40°±5.77°, 19.96°±5.53°, 20.56°±6.20°, and 21.46°±5.32° for five different areas of the device. This shows comparatively larger tilt angles with larger standard deviations for the non-sublimated device in comparison to the sublimated device. Also, we found that the sublimated device shows smaller value of the average angle differences between neighboring measurements points with smaller deviation in all the measured areas compared to those for the non-sublimated device. These results confirm uniformity over a large area of the active layer in both devices.


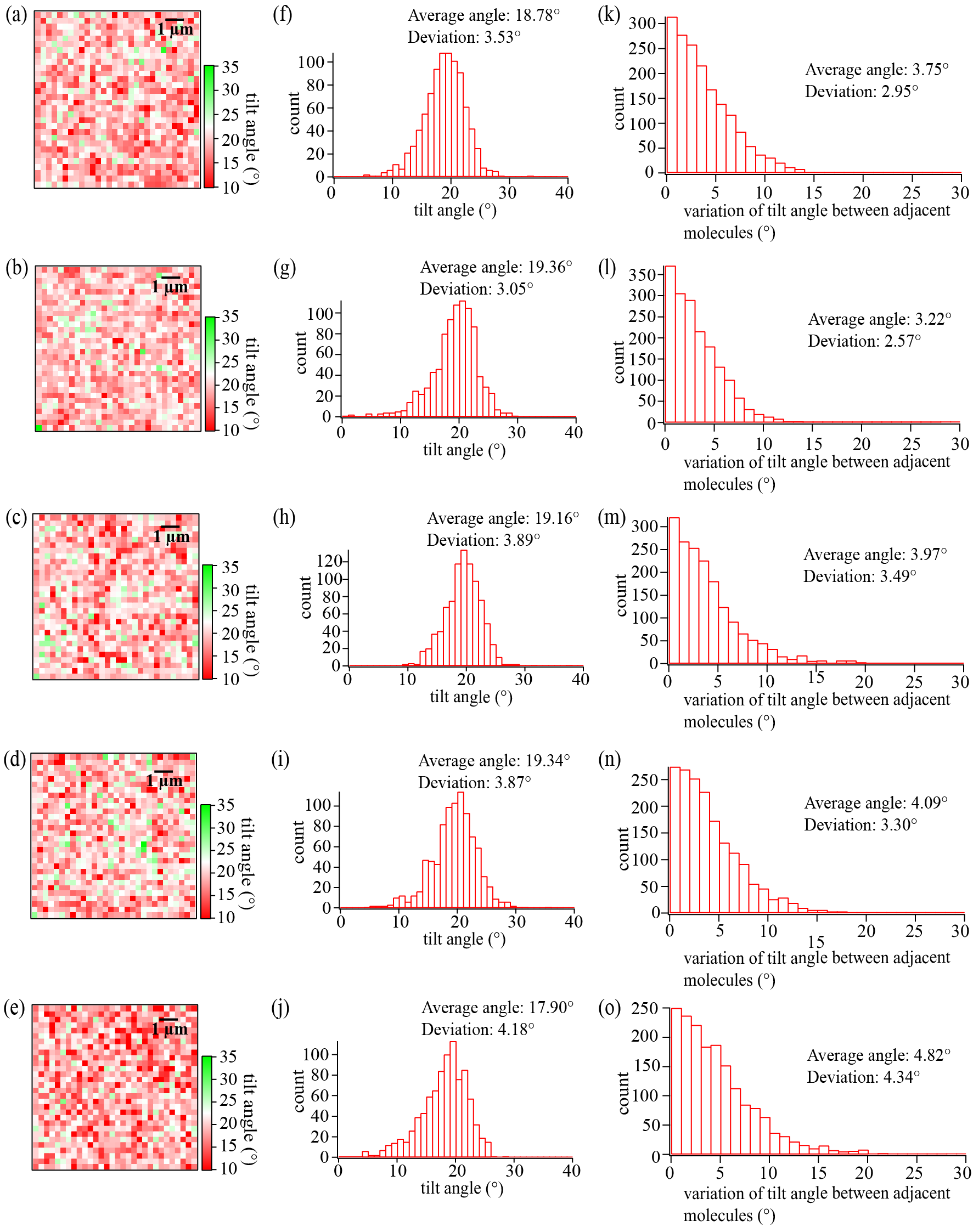


**Figure S1.** (**a**)-(**e**) show the molecular orientation distribution for five different areas of the sublimated pentacene transistor device. Histograms (**f**)-(**j**) show the corresponding tilt angle variation for the same five positions, and histograms (**k**)-(**o**) show the variation of the tilt angle between adjacent positions, which is separated by a few hundred nanometers in the device.


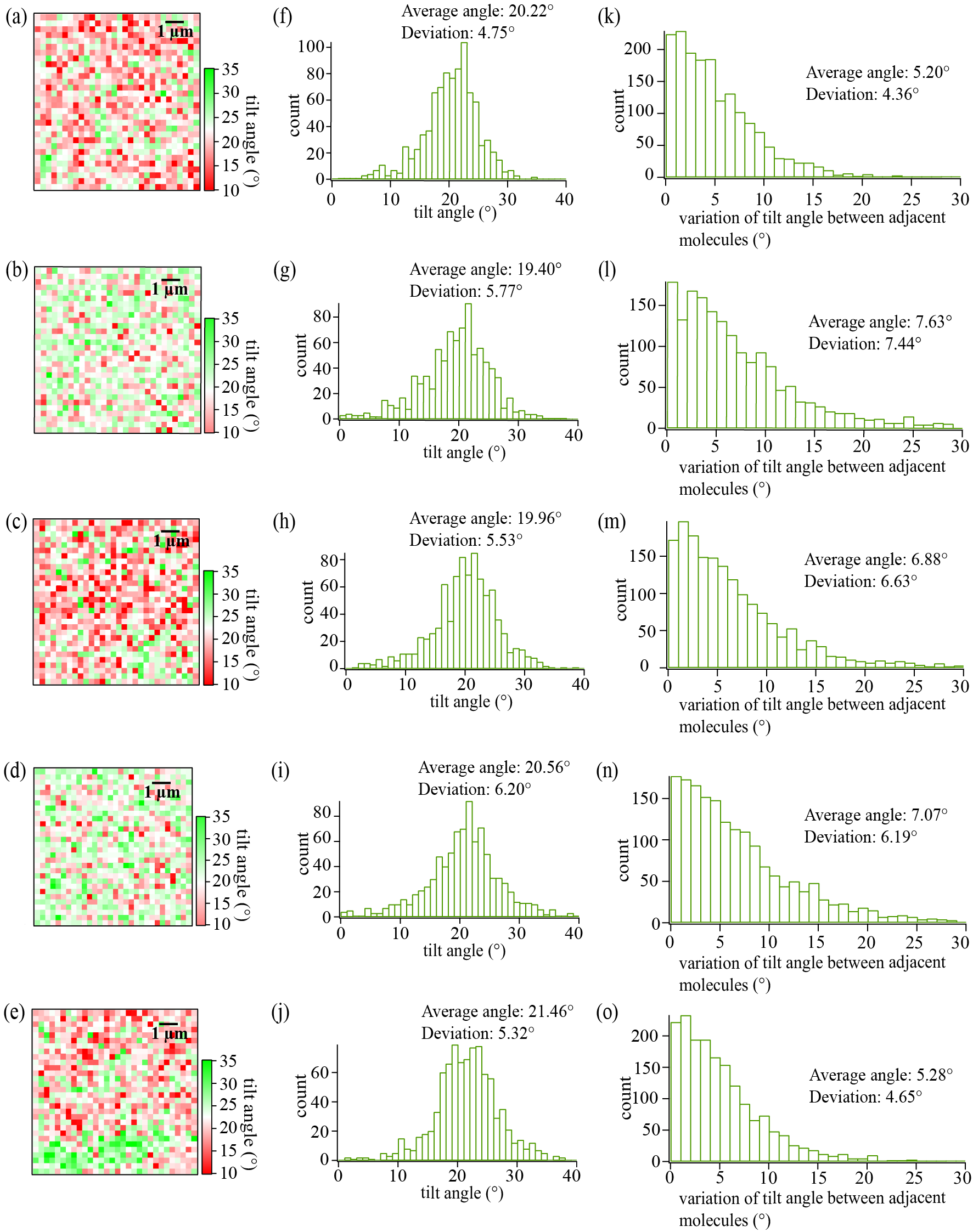


**Figure S2.** (**a**)-(**e**) show the molecular orientation distribution for five different areas of the non-sublimated pentacene transistor device. Histograms (**f**)-(**j**) show the corresponding tilt angle variation for the same five positions, and histograms (**k**)-(**o**) show the variation of the tilt angle between adjacent positions, which is separated by a few hundred nanometers in the the device.
